# Surgical Approach and Management Strategies in a Pediatric Cardiovascular Surgery Clinic During the COVID-19 Outbreak

**DOI:** 10.21470/1678-9741-2020-0614

**Published:** 2022

**Authors:** Ergin Arslanoğlu, Mehmet Emirhan Işık, Kenan Abdurrahman Kara, Nihat Çine, Eylem Tunçer, Hakan Ceyran

**Affiliations:** 1 Department of Pediatric Cardiovascular Surgery, Kartal Kosuyolu Yuksek Ihtisas Egitim ve Arastirma Hastanesi, Istanbul, Turkey.; 2 Department of Infectious Disease, Kartal Kosuyolu Yuksek Ihtisas Egitim ve Arastirma Hastanesi, Istanbul, Turkey.

**Keywords:** Congenital Heart Disease, Cardiac Surgical Procedures, Covid-19, Real Time Polymerase Chain Reaction, Delivery of Health Care, Early Diagnosis, Child

## Abstract

**Introduction:**

The coronavirus disease 2019 (COVID-19) pandemic has required changes in the management of pediatric cardiac surgery. We would like to share the patient treatment and surgical management strategies employed in our Pediatric Cardiovascular Surgery Clinic during the COVID-19 pandemic.

**Methods:**

A total of 112 patients were followed up in our clinic between 11.03.2020 and 02.07.2020. Their mean age was 1,118 (4-5,740) days. Management and treatment were performed by our pediatric heart team (pediatric cardiac anesthetists, general pediatricians, pediatric cardiologists, pediatric cardiac surgeons, and an infectious diseases specialist). We prepared new protocols and a surveillance system specific to the pandemic to prevent in-hospital transmission and reduce postoperative mortality and morbidity; our operations were performed according to these protocols. All decisions pertaining to the operation timing and treatment strategy of our COVID-19-positive patients were made by the same team.

**Results:**

During the study period, a total of 112 patients, 69 boys and 43 girls, were hospitalized in our clinic. A total of 333 COVID-19 real-time polymerase chain reaction tests were performed on patients and accompanying persons; positive results were found in three patients and two accompanying individuals.

**Conclusion:**

By employing new protocols and a surveillance system throughout the healthcare system, we think that early diagnosis and treatment of the pediatric congenital heart disease population, which is susceptible to infections, can continue unperturbed. This and similar approaches can increase postoperative success and prevent transmission in the pediatric population - which are frequently COVID-19 asymptomatic.

**Table:** 

Abbreviations, acronyms & symbols			
ASD	= Atrial septal defect	OR	= Operating room
AV	= Atrioventricular	Pa	= Posteroanterior
AVSD	= Atrioventricular septal defect	PA	= Pulmonary atresia
BT	= Blalock-Taussig	PAPVR	= Partial anomalous pulmonary venous return
CHD	= Congenital heart disease	PCR	= Polymerase chain reaction
CMP	= Cardiomyopathy	PDA	= Patent ductus arteriosus
COVID-19	= Coronavirus disease 2019	PPE	= Personal protective equipment
CRP	= C-reactive protein	PS	= Pulmonary stenosis
CT	= Computed tomography	RT-PCR	= Real-time polymerase chain reaction
CVE	= Cerebrovascular event	TGA	= Transposition of the great arteries
ECMO	= Extracorporeal membrane oxygenation	TOF	= Tetralogy of Fallot
HCP	= Healthcare professionals	VSD	= Ventricular septal defect
ICU	= Intensive care unit	WBC	= White blood cell

## INTRODUCTION

The first coronavirus disease 2019 (COVID-19) case in the world was reported in Wuhan, China, in December 2019. And the first case in Turkey was recorded on March 11, 2020, coinciding with the date on which the World Health Organization (or WHO) declared the outbreak as a pandemic^[1^,^2]^. COVID-19 poses a serious risk for children with congenital heart disease (CHD), who are vulnerable to pulmonary infections due to comorbidities such as pulmonary congestion^[3]^.

Information on treatment methods and strategies for infants with CHD during the COVID-19 outbreak is limited^[4]^. It is well-established that 25% of CHD cases require a surgical intervention within the first year of their lives^[5]^. Even during the COVID-19 outbreak, CHD operations should be performed as soon as possible.

We aimed to explain our approach to patient management at our Pediatric Cardiovascular Surgery Clinic during the COVID-19 outbreak.

## METHODS

The present study is a retrospective, observational, single-center case series. The study included 112 inpatients who were treated and followed up at the Pediatric Cardiovascular Surgery Clinic of Health Sciences University, Koşuyolu High Education Training and Research Hospital, from March 11, 2020, to July 2, 2020 — during the COVID-19 outbreak. The patients’ age, gender, weight, diagnoses, previous surgeries and invasive procedures, clinical status in terms of COVID-19, polymerase chain reaction (PCR) tests, and imaging results were recorded. During the COVID-19 outbreak, elective cases were postponed in line with the directives of the Ministry of Health. Only emergency cases were admitted to our clinic. All inpatients were clinically evaluated by the local pandemic committee and nasopharyngeal, oropharyngeal, and lower respiratory tract samples were obtained from all patients and accompanying individuals for COVID-19 testing with real-time PCR (RT-PCR). Permissions were obtained from the Ministry of Health and our hospital’s ethics committee for the present study (2020-11-377).

### Case Selection

Hospitalization decisions were made by the local pandemic committee, consisting of a pediatric cardiovascular surgeon, pediatric cardiologist, pediatrician, infectious diseases specialist, and anesthesiologist in our hospital, in line with the Pandemic Action Plans constituted by the Ministry of Health and the hospital management. Elective case admission was terminated after our hospital was declared as a “pandemic hospital” by the Ministry of Health. However, due to the emergency of pediatric cardiovascular surgeries, the committee decided to continue performing these surgeries. The emergency situations of the patients were determined by clinical, laboratory, and radiological evaluations. Emergency cases were operated on by using personal protective equipment (PPE) without waiting for COVID-19-specific laboratory and imaging results.

All patient rooms were remodeled to accommodate a single patient. Patients referred to our clinic from external centers were required to apply with their COVID-19 RT-PCR test results. Operations of positive COVID-19 patients were postponed when deemed necessary by the committee according to their clinical conditions, and these patients received COVID-19 treatment.

**Fig. 1:**
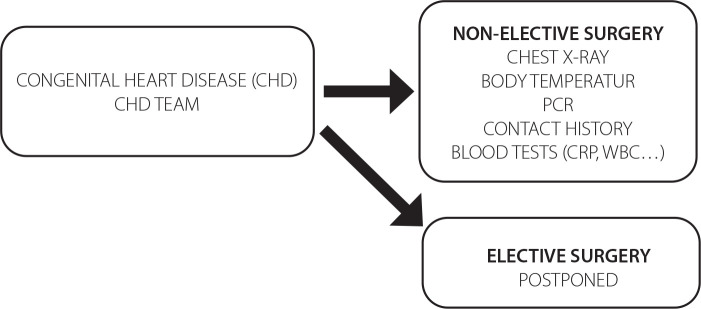
Algorithm for preoperative patients. CRP=C-reactive protein; PCR=polymerase chain reaction; WBC=white blood cell

Algorithms for all surgery and patient care processes were developed or redesigned by the pandemic committee of our hospital (Figure 1). COVID-19 RT-PCR tests were routinely applied to all cases before surgery. Posteroanterior (Pa) chest radiography or computed tomography (CT) of the chest were performed in clinically suspected cases. Surgical masks were provided to all patients and their attendants during the hospitalization period. All healthcare professionals (HCP) were given training by infectious diseases specialists on protection from COVID-19. In addition, in our clinic, support personnel, ward personnel, and intensive care personnel were trained on hand hygiene, use of PPE, and the clinical features of COVID-19 patients.

The patients and their attendants were informed about the use of PPE, hand disinfection, social distancing, and the COVID-19-related rules that were to be followed in the clinic. Common areas were closed. Hospital cleaning and disinfecting procedures were reevaluated, and their frequency were increased. Triage was applied at the entrances and exits of the hospital and individuals with COVID-19 symptoms were examined in isolated areas. Everyone entering the hospital, including all personnel, underwent body temperature measurements at the entrances of the facility. All patients and their attendants were informed about the pandemic and modified informed consent forms — prepared according to COVID-19-related regulations, with additional clauses and explanations — were signed before surgery.

To reduce the risk of contamination, all HCP switched to the shift system. Telephone and video conference calls were made on certain days of the week instead of committee meetings. All necessary PPEs were provided to all HCP. Online trainings were held for HCP on latest information regarding COVID-19.

After transportation of patients into the operating room (OR), all OR personnel wore PPE. Entries and exits to the OR were limited as much as possible. FFP2/FFP3 masks, fluid-proof gowns, and face shields or goggles were used during intubation and surgery. Surgeons wore protective gloves during surgery. Intubations were performed with video-supported laryngoscopes in intubation cabinets. Aspiration was performed with closed aspiration systems. Virus filters were placed in the intubation tubes during the transport of intubated patients.

COVID-19 RT-PCR tests of all patients with postoperative fever were repeated. Required treatments were planned for these patients. Also, 20 mg/kg vitamin C and vitamin D were added to the postoperative treatment regimen of all patients.

## RESULTS

During the study period, a total of 112 patients, 69 boys and 43 girls, were hospitalized in our clinic. COVID-19 RT-PCR test was performed to all patients before admission. The mean age of the patients was 1,118 (4-5,740) days. The mean length of stay in the intensive care unit (ICU) was 7.51 (0-66) days, the mean length of stay in the ward was 4.41 (1-39) days, and the mean duration of mechanical ventilation was 3.43 (0-101) days.

A total of 333 COVID-19 RT-PCR tests were performed on patients and accompanying persons; positive results were found in three patients and two accompanying individuals. While the COVID-19 RT-PCR test result was positive for the patient of one accompanying person, the result of the patient of the other was negative (Table 1). None of the HCP in our clinic had a positive COVID-19 RT-PCR test result.

**Table 1:** Patients and their accompanying persons with positive COVID-19 RT-PCR test results.

	Diagnosis	Procedure	Status	RT-PCR (patient/accompanying person)	ICU (days)	Ward (days)
1	CMP	Medical treatment	Died	+/-	1	2
2	Coarctation of the aorta	Repair of the coarctation	Died	+/+	0	5
3	ASD-VSD-PA	Central shunt	Discharged	+/-	7	3
4	Hypoplastic left heart syndrome	Norwood stage 1	Died	-/+	23	0

ASD=atrial septal defect; CMP=cardiomyopathy; COVID-19=coronavirus disease 2019; ICU=intensive care unit; PA=pulmonary atresia; RT-PCR=real-time polymerase chain reaction; VSD=ventricular septal defect

During the study period, 83 patients were operated in our clinic, 21 patients underwent invasive procedures (angiography, etc.), and eight patients received medical treatment. Tetralogy of Fallot operations were performed on 13 patients. Ventricular septal defect (VSD) closure was performed in 21 patients. Balloon valvuloplasty was performed in nine patients with pulmonary stenosis. Rastelli procedure was performed in three patients with VSD + pulmonary atresia (PA). Glenn procedure was performed in eight patients, Kawashima procedure in two patients, Fontan procedure in two patients, and central shunt procedures in two patients. Atrioventricular septal defect was repaired in two patients. Arterial switch was performed in three patients with transposition of the great arteries. Norwood stage 1 operation was performed in three patients, surgery of coarctation of the aorta in three patients, pulmonary band surgery in five patients, arcus reconstruction in two patients, and pacemaker change in four patients. Atrial septal defect (ASD) + partial anomalous pulmonary venous return was repaired in three patients. One patient’s ASD and one patient’s patent ductus arteriosus (PDA) were closed. Aortic valve stenosis was repaired in five patients. PDA surgery was performed in three patients. Diagnostic coronary angiography was performed in 10 patients. Medical follow-up was performed in four patients with cardiomyopathy (CMP), two with pericarditis, and two with congestive heart failure. Complications were as follows: complete atrioventricular block in two patients, cerebrovascular event in one patient, prolonged intubation in 12 patients, need for extracorporeal membrane oxygenation (ECMO) in seven patients, bleeding revision in five patients, and eight patients died. The operations performed in the patients and the complications are summarized in Table 2.

**Table 2:** Operations and complications during the COVID-19 outbreak.

Operation	Case	Discharge	Death	Complication
TOF	13	13	0	ECMO (1), prolonged intubation (1)
VSD	21	21	0	Complete AV block (2)
PS-balloon valvuloplasty	9	9	0	0
Rastelli (VSD+PA)	3	2	1	Bleeding revision (1)
Glenn	8	8	1	ECMO (1), prolonged intubation (3)
Kawashima	2	2	0	0
Fontan	2	1	1	ECMO (1), prolonged intubation (1)
Central shunt	2	2	0	0
AVSD repair	2	2	0	0
TGA - arterial switch	3	3	0	ECMO (1), prolonged intubation (1), bleeding revision (1)
Norwood stage 1	3	1	2	ECMO (1), prolonged intubation (3) bleeding revision (2)
Coarctation of the aorta	3	3	0	0
Pulmonary band	5	4	1	Prolonged intubation (1)
Arcus reconstruction	2	2	0	Bleeding revision (1)
Pacemaker change	4	4	0	0
ASD + PAPVR	3	3	0	0
ASD closure with device	1	1	0	0
Aortic valve stenosis	5	4	1	ECMO (2), CVE (1), prolonged intubation (2)
PDA closure (3 with surgery, 1 with device)	4	4	0	0
Diagnostic angiography	10	10	0	0
Pericarditis - medical	2	2	0	0
Congestive heart failure - medical	2	2	0	0
Dilate CMP - medical	4	3	1	0

ASD=atrial septal defect; AV=atrioventricular; AVSD=atrioventricular septal defect; CHD=congenital heart disease; COVID-19=coronavirus disease 2019; CVE=cerebrovascular event; ECMO=extracorporeal membrane oxygenation; PA=pulmonary atresia; PAPVR=partial anomalous pulmonary venous return; PS=pulmonary stenosis; TGA=transposition of the great arteries; TOF=tetralogy of Fallot; VSD=ventricular septal defect

### Case 1

**Fig. 2:**
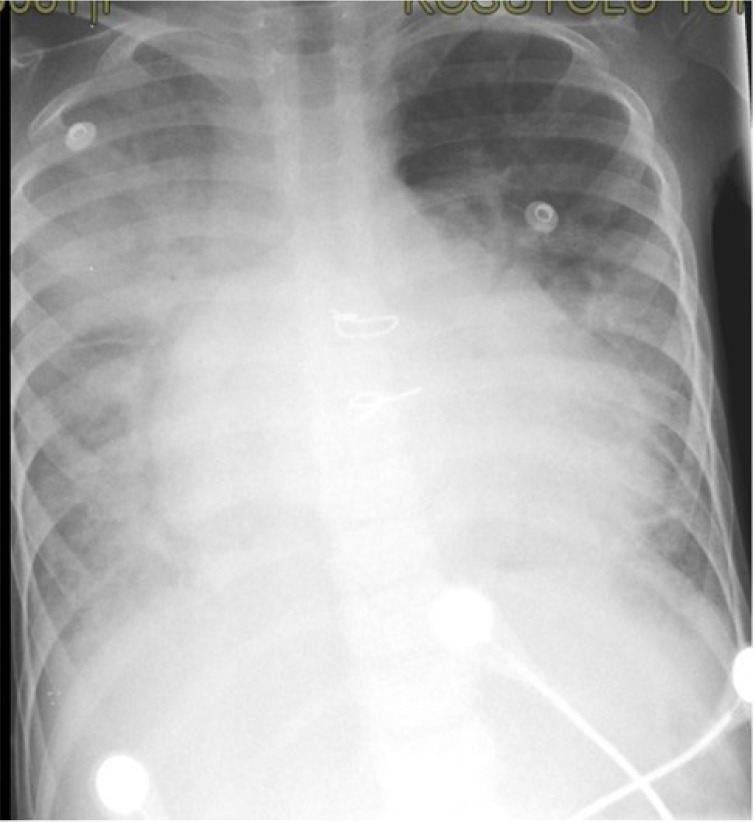
Tele-imaging of the case with dilated cardiomyopathy who is positive for coronavirus disease 2019.

A six-year-old girl with dilated CMP, who was admitted to the emergency room with symptoms of heart failure and fever, was hospitalized. The patient’s COVID-19 RT-PCR test was positive, and widespread infiltrations were detected in her tele-imaging (Figure 2). On the second day after the patient was hospitalized, her general condition deteriorated, and eventually, she died.

### Case 2

In this case, the patient was a 33-day-old boy who had aortic coarctation and VSD. The child and his mother were both found to be positive with COVID-19 RT-PCR tests. The scheduled surgery was postponed with the decision of the committee, and the patient and the mother were treated for COVID-19. In the medical follow-up, the patient was admitted to ICU due to rapid deterioration in left ventricular function and development of heart failure. Despite medical treatment, his general condition deteriorated, and eventually, he died.

### Case 3

The Blalock-Taussig (BT) shunt operation was performed on a patient who applied to our clinic with diagnoses of right ventricular hypoplasia, PA, VSD, and ASD. The patient had postoperative fever in the ICU and the COVID-19 RT-PCR test was found to be positive. After treatment for COVID-19, the control COVID-19 RT-PCR test was found to be negative. The patient, who was intubated for 72 hours, was extubated after clinical improvement, and was observed during follow-up. The patient was monitored in the ICU for seven days and in the ward for three days, being discharged after recovery.

### Case 4

**Fig. 3:**
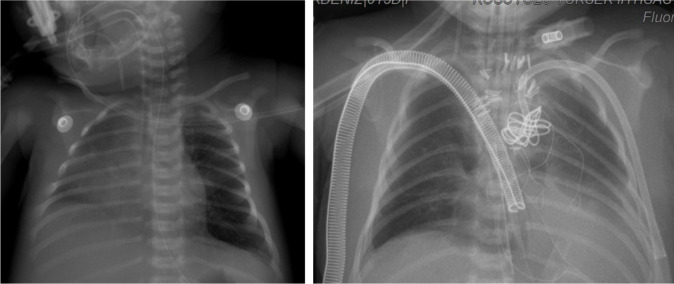
Preoperative and postoperative tele-imaging of the patient whose coronavirus disease 2019 real-time polymerase chain reaction (RT-PCR) test was negative but whose mother’s RT-PCR was positive and who underwent Norwood stage 1 operation.

An intubated newborn with hypoplastic left heart syndrome was admitted to our clinic from another center. The patient’s COVID-19 RT-PCR test was found to be negative, and no pneumonia findings were identified in the Pa chest X-ray and CT, but the mother’s COVID-19 RT-PCR test was found to be positive (Figure 3). Norwood stage 1 operation was performed on the patient. The control COVID-19 RT-PCR test in the postoperative period was also found to be negative. The patient was monitored on ECMO with intubation for 23 days in the ICU. The patient, whose general condition deteriorated during follow-up, died due to sepsis caused by a non-coronavirus cardiac infection.

## DISCUSSION

The COVID-19 outbreak is one of the greatest health events worldwide in the last centuries and it has significantly increased the burden on healthcare systems. Although clinical symptoms of COVID-19 are generally less severe in children than adult patients, young children and especially infants with CHD are highly susceptible to this infection^[6^,^7]^. Infants with CHD are at high risk in terms of COVID-19 because they need to stay in the hospital environment for a long time and are in close contact with their families. The treatment strategies and methods to be applied to CHD cases should be determined by considering both the general condition of the patient and the restrictions of the healthcare system. Since it is necessary to alleviate the burden on the healthcare system while also employing necessary precautions against COVID-19, the risk of mortality and morbidity in these high-risk operations may increase. In a study by Stephen et al.^[8]^, it was stated that CHD operations were planned by a team consisting of a pediatric cardiovascular surgeon, pediatric cardiologist, and pediatrician, and in consultation with the patient’s family.

It has been reported that COVID-19 cases in the pediatric age group generally have a relatively mild course of the disease^[9]^. In a retrospective study by Dong et al.^[10]^, only one of the 2,135 pediatric COVID-19 patients died, and the incidence of severe symptoms was found to be lower than in adults. Potent natural immunity, frequent vaccination, and the high regeneration capacity of the pediatric alveolar epithelium may be among the reasons of this situation. Another reason is that children have fewer risk factors, such as comorbidities, smoking, and obesity. Nevertheless, COVID-19 is relatively severe in babies with CHD, possibly due to the presence of congenital immunodeficiency^[11]^.

In the present study, a total of 333 COVID-19 RT-PCR tests were performed on patients and accompanying persons, which revealed COVID-19 positivity in three patients and two accompanying individuals. Levy et al.^[12]^ had previously recommended obtaining oropharyngeal, nasopharyngeal, or lower respiratory tract samples for COVID-19 RT-PCR tests in patients scheduled for pediatric cardiovascular surgery and their parents. They also suggested that, in COVID-19 positive cases, surgeries should be postponed for up to 14 days if there is no emergency.

In addition to clinical evaluation and laboratory analyses, Pa chest X-ray is preferred as a radiological technique for the diagnosis of COVID-19. When the diagnosis cannot be clarified, especially in children, Pa chest X-ray have been recommended with respect to the level of radiation exposure^[12]^.

In a study conducted by Guo et al.^[13]^, myocardial damage was found in 27.8% of adult COVID-19 patients. The relationship between COVID-19 and myocardial damage may have a role in the rapid deterioration of the general condition of the patient with dilated CMP in our study.

In our 33-day-old patient with aortic coarctation and VSD, echocardiography revealed normal left ventricular function. However, while under COVID-19 treatment, left ventricular function deteriorated, and the patient died. Left ventricular functions are not expected to deteriorate so quickly in patients with aortic coarctation^[13]^. We think that this situation was associated with COVID-19.

The COVID-19 treatment of our patient with right ventricular hypoplasia was successfully completed and right BT shunt was performed after the COVID-19 RT-PCR test was negative. The patient, who received mechanical ventilation for three days during a seven-day stay in ICU, was then transferred to the ward for three days and was finally discharged.

## CONCLUSION

In conclusion, a surveillance system that can be applied throughout the hospital and healthcare system can guide the early diagnosis and treatment of pediatric cases that are often asymptomatic in terms of COVID-19 and patient groups that are more susceptible to viral infections. In addition, since individuals with asymptomatic or mild COVID-19 play a major role in the spread of COVID-19, such surveillance systems can contribute to more meticulous implementation of social distancing and the application of routine protective measures for people of all ages to slow the spread of the virus.

**Table:** 

Authors' roles & responsibilities	
EA	Substantial contributions to the conception or design of the work; or the acquisition, analysis, or interpretation of data for the work; drafting the work or revising it critically for important intellectual content; final approval of the version to be published
MEI	Substantial contributions to the conception or design of the work; or the acquisition, analysis, or interpretation of data for the work; final approval of the version to be published
KAK	Drafting the work or revising it critically for important intellectual content
NÇ	Final approval of the version to be published
ET	Final approval of the version to be published
HC	Final approval of the version to be published
